# *Bergenia pacumbis* (Buch.-Ham. ex D.Don) C.Y.Wu & J.T.Pan: A Comprehensive Review on Traditional Uses, Phytochemistry and Pharmacology

**DOI:** 10.3390/plants11091129

**Published:** 2022-04-21

**Authors:** Apurba Gohain, Ajay Sharma, Hirok Jyoti Gogoi, Raymond Cooper, Ramandeep Kaur, Gulzar Ahmad Nayik, Ayaz Mukarram Shaikh, Béla Kovács, Franklin Ore Areche, Mohammad Javed Ansari, Nadiyah M. Alabdallah, Ammar AL-Farga

**Affiliations:** 1 Department of Chemistry, Assam University Silchar, Dorgakona, Silchar 788011, Assam, India; apurbaburagohainapurba998@gmail.com; 2 University Centre for Research and Development, Chandigarh University, Mohali 140413, Punjab, India; sharmaajay9981@gmail.com; 3 Department of Chemistry, Chandigarh University, Mohali 140413, Punjab, India; 4 Department of Life Science and Bioinformatics, Assam University Diphu Campus, Diphu 782460, Assam, India; hgogoi149@gmail.com; 5 Department of Applied Biology and Chemical Technology, The Hong Kong Polytechnic University, Hong Kong, China; rcooperphd@aol.com; 6 Department of Chemistry, Punjab Agricultural University, Ludhiana 141004, Punjab, India; ramanhunjan@pau.edu; 7 Department of Food Science & Technology, Government Degree College Shopian, Shopian 192303, Jammu and Kashmir, India; gulzarnaik@gmail.com; 8 Institute of Food Science, University of Debrecen, 138 Böszörményi St., 4032 Debrecen, Hungary; kovacsb@agr.unideb.hu; 9 Professional School of Agroindustrial Engineering, National University of Huancavelica, Huancavelica 09001, Peru; franklin.ore@unh.edu.pe; 10 Department of Botany, Hindu College Moradabad, (Mahatma Jyotiba Phule Rohilkhand University Bareilly), Moradabad 244001, Uttar Pradesh, India; mjavedansari@gmail.com; 11 Department of Biology, College of Science, Imam Abdulrahman Bin Faisal University, P.O. Box 1982, Dammam 31441, Saudi Arabia; nmalabdallah@iau.edu.sa; 12 Department of Biochemistry, College of Sciences, University of Jeddah, P.O. Box 34, Jeddah 21959, Saudi Arabia; amalfarga@uj.edu.sa

**Keywords:** pashanbheda, *Bergenia ligulata*, medicinal plant, secondary metabolites, polyphenols, biological potential

## Abstract

The influence of medicinal plants on humanity spans time immemorial. These plants are also used at present with local and tribal peoples for the cures of various illnesses. Nature has produced an immense number of medicinal plants, which directly or indirectly help to treat various ailments and have numerous applications in the fields of pharmaceuticals, agriculture, food flavors and preservatives, aromas, and cosmetics. *Bergenia pacumbis* (Buch.-Ham. ex D.Don) C.Y.Wu & J.T.Pan (synonym: *Bergenia ligulate* Engl.), is an important medicinal plant belonging to the Saxifragaceae family, and not to be confused with *Bergenia ciliata* (Haw.) Sternb., and is popularly known as Pashanbheda (meaning to dissolve the kidney stone). This plant is a rich source of secondary metabolites (SMs) such as coumarins, flavonoids, benzenoids, lactones, tannins, phenols, and sterols, which make this plant a highly valued medicinal herb with a broad spectrum of pharmacological activities such as anti-urolithic, antioxidant, anti-viral, free radical scavenging, antidiabetic, anti-hepatotoxic, diuretic, antipyretic, anti-oxaluria, anti-tumour, antibacterial, antifungal, anti-inflammatory, antimicrobial, and cardioprotective. This review summarizes traditional uses and offers up to date data for future research on *B. pacumbis*.

## 1. Introduction

Medicinal plants are a significant gift of nature to humankind, which help us cure many diseases and have many applications in cosmetics, dyes, beverages, food flavors, and preservatives [1]. The herbal drugs obtained from medicinal plants are usually cost effective, easily accessible, and eco-friendly in nature, this leads to their wide acceptance across the globe. Earlier evidence of the use of medicinal plants has been recorded for more than five thousand years in traditional literature such as classical Indian texts(Charak Samhita, Atherveda, Rigveda, and Sushruta Samhita), Chinese, Egyptian, Greek, Roman, and Syrian literature [2,3]. A survey conducted by the World Health Organization (WHO) reveals that about 80% of the world’s population depends upon the use of plant-mediated herbal medicines for the treatment of various ailments. WHO has also recognized the existence of therapeutic practices of herbal plants over hundreds of years before the onset of the development of modern medicines [4,5]. According to essential guidelines prescribed by WHO, quality, identity, and non-toxicity are three significant characteristics for any herbal medicine. Due to those guidelines, medicinal plants that do not have any botanical identity are considered controversial drugs [3,4]. However, plant-based bioactive drugs can act as a source of valuable medicine in modern times, due to reduced toxicity and better conjugation with biological systems [6]. Various secondary metabolites (SMs) present in medicinal plants are well known for their enormous curative potential against various disorders, such as haemorrhoids, memory loss, osteoporosis, diabetes, wounds, cancer, HIV/AIDS, Alzheimer’s, malaria, pain, and liver dysfunction [7].

To date, numerous plant families/species are recognized in the eastern Himalayas for their bioactive SMs [8,9]. Saxifragaceae is one of the most significant plant families, with 33–35 genera and 640 known species worldwide. The Saxifragaceae family has significant diversification from both ethnobotanical and medicinal values compared to many other plant families [10,11,12]. People have used the plants of this family to cure many ailments since ancient times [3,10,11,12,13]. Various plant species are distributed worldwide, from cold northern areas to temperate regions, widespread over East Asia or Western North America, and Western Europe and South America [11,12,14]. This plant family is distributed in the temperate Himalayas in India, mainly from Kashmir to Nepal to the Khasia hills [3,10,11,12,13,14,15].

Among 33–35 genera of the family Saxifragaceae, species of the *Bergenia* genus are the most explored and studied for their bioactive SMs composition and medicinal uses [13,16]. This genus is native to central Asia, from China to Afghanistan and the Himalayan region. The general botanical characteristics of *Bergenia* genus plants are perennial herbs, up to 35 cm tall. Stems are usually short, thick, fleshy, and procumbent. Leaves are ovate and about 5–15 cm in length. The upper and lower surface of the leaves are hairy. Flowers are white, pink, or purple. The sepals are 7 mm long, petals are 10 × 4 mm, with two carpels, filaments are 1 cm long, the styles are 7 mm long, and the capsules are 13 × 6 mm. The seeds are brown elongated up to 1 mm long. The inflorescence is a raceme or corymbose type. The rhizome is solid, barrel, and cylindrical [3,5,10,11,12,14,15].

Within the *Bergenia* genus, *Bergenia* × *beesiana* C.K. Schneid., *Bergenia yunnanensis*, *Bergenia ugamica* V.N.Pavlov, *Bergenia biflora* Moench, *Bergenia tianquanensis* J.T. Pan, *Bergenia ciliata* (Haw.) Sternb., *Bergenia thysanodes* (Lindl.) C.K. Schneid., *Bergenia cordifolia* (Haw.) Sternb., *Bergenia* × *spathulata* Nagels ex Guillaumin, *Bergenia coreana* Nakai, *Bergenia* × *smithii* Engl., *Bergenia crassifolia* (L.) Fritsch, *Bergenia scopulosa* T.P.Wang, *Bergenia emeiensis* C.Y.Wu ex J.T.Pan, *Bergenia delavayi* (Franch.) Engl., *Bergenia* × *schmidtii* (Regel) Silva Tar., *Bergenia purpurascens* (Hook.f. & Thomson) Engl., *Bergenia stracheyi* (Hook.f. & Thomson) Engl., *Bergenia pacumbis* (Buch.-Ham. ex D. Don) C.Y. Wu & J.T. Pan (synonym: *Bergenia ligulata* Engl.), *Bergenia crassifolia* var. pacifica (Kom.) Kom. ex Nekr., *Bergenia hissarica* Boriss., *Bergenia* × *ornata* Stein and *Bergenia orbicularis* Stein, are some essential species that have been well known for their enormous medicinal values [5,13]. The bioactive compounds obtained from the different species of the *Bergenia* genus belongs to various classes of SMs such as alkaloids, terpenoids, phenolics, and steroids, which have been known for various pharmacological effects in human beings and hence are used as drugs [3,17,18,19]. Bergenin, tannic acid, stigmasterol, β- sistosterol, catechin, afzelechin, (+)-(6S)-parasorbic acid, phytol, caryophyllene, damascenone, β-eudesmol, 1,8-cineole, 3-methyl-2-buten-1-ol, isovaleric acid, (Z)-asarone, terpinen-4-ol, paashaanolactone, arbutin, and gallic acid are the key bioactive SMs obtained from various species of the *Bergenia* genus [3,18,19,20]. These are mainly used in the perfumery, pharmaceuticals, flavoring, and cosmetics industries [17]. The *Bergenia* genus contains various classes of SMs, viz., alkaloids, terpenoids, phenolics, and steroids, which have been known for various pharmacological effects in human beings and hence are used as drugs [3,18,19,21].

*Bergenia pacumbis* is recognised as an endangered or threatened and vulnerable medicinal plant species using the new IUCN (International Union for Conservation of Nature) criteria, so conservation of this plant is necessary. This article provides detailed procedures for the cultivation of *B. pacumbis*, which is very important and was lacking in previously published review papers [3,5,13,19,21,22]. Thus, the efficient strategies for usage, conservation, protection initiatives, and effective agro-techniques of the species are instantly required. *Bergenia pacumbis* is a rich source of a variety of naturally occurring bioactive secondary metabolites, but the extracts and naturally occurring bioactive secondary metabolites of this plant have not as yet been fully explored both in-vivo and in-vitro model studies [3,5,21]. Therefore, this plant has strong potential for further impact in the field of phytochemistry and pharmacology. Further, due to the lack of proper taxonomic profiling, people often misidentify *B. pacumbis* with other species of the same genus due to the misunderstanding in the morphology of the species. This article provides brief information about the morphology of the *B. pacumbis* and is helpful for the correct identification and authentication of this species. It is expected that this article will encourage the researcher worldwide to explore more and more about this medicinal plant. As this species is endangered, it is more important to study this plant thoroughly to know its full potency, and there is a need to find a better way to cultivate this plant. This review article summarizes the available literature on *B. pacumbis* regarding its botanical description, morphology, traditional uses, cultivation procedure, phytochemicals, pharmacological activities, and patents filed on this plant. It provides up to date data for future research in the potential manufacture of new drugs and further clinical studies.

## 2. *Bergenia pacumbis* (Buch.-Ham. ex D.Don) C.Y.Wu & J.T.Pan (Synonym: *Bergenia ligulata*)

*Bergenia pacumbis* is one among the most vital species of the *Bergenia* genus, popularly known as the stone flower, the stone breaker, and pashanbheda [23]. It is a perennial herb rich in many phytochemical constituents, which mainly occur in temperate regions of the Himalayas from Kashmir to Bhutan in the Khasia Hills at 1800–5100 m [3,22,24], usually growing in rocky areas and cliffs. It acts as a prime storehouse of bioactive SMs like β- sitosterol, tannic acid, stigmasterol, gallic acid, bergenin, (+)-afzelechin, (+)-afzelechin tetraacetate, (+)-5,7,4ؘ-trimethyl afzelechin, (+)-tetramethoxyafzelechin, and (+)-3-acetyl-5,7,4ؘ-trimethoxyafzelechi [3,4,21,22]. This plant is regarded as a highly valued medicinal herb in traditional Nepalese, Indian, and Chinese medicine systems. It is mainly used for the cure of pulmonary diseases, coughs, to increase immunity in humans, to stop bleeding, and to dissolve bladder or kidney stones [3,21,24]. *Bergenia pacumbis* also possesses wide clinical applications such as to treat psoriasis [3,5], airway mucus hypersecretion and asthma, kidney disorders [3,21,25,26,27], the inhibition of blood cholesterol level absorption in the intestine [28], the inhibition of CaC_2_O_4_ crystal formation, crystal aggregation, crystal deposition in renal tubules, the inhibition of calcium and phosphate accumulation, the inhibition of *α*-glucosidase [29,30], it suppresses arachidonic acid metabolism and production of cytokine in human monocytes [31], reduces the urine output and dieresis production of urea [32], and shows hypermagnesemia activity. Moreover, this plant has a wide spectrum of pharmacological effects such as anti-histamines, anti-tissusive, insecticidal, anti-convulsant in valerian, anti-oxidant [3,20,33,34], anti-viral against influenza virus [35,36], antidiabetic [37], hypoglycemic, diuretic, anti-oxaluria, and has cytotoxic activity [3,24,37,38,39].

Although this plant is commonly grown and traditionally used by local people of the northern Himalayan region, Kashmir, Bhutan, and Khasia hills to cure various illnesses, its clinical applications have not been explored to their full potential. Furthermore, the plant is not used as a whole, instead its individual part demonstrates engaging pharmacological activities. The presence of many SMs implies a high chance to manufacture drugs, which can benefit humanity.

### 2.1. Methodology

Our information gathering started with the search of relevant literature on *B. pacumbis*, and all its synonyms were confirmed through the plant database (http://www.theplantlist.org/tpl1.1/record/kew-2674823, accessed on 20 April 2022,). This review article discusses medicinal uses, phytochemistry, pharmacological effects, and toxicity of *B. pacumbis* from various databases such as Google Scholar, Web of Science, Science Direct, Scopus, and PubMed. In total, 102 articles were reviewed. In this review, only the articles published in the English language were taken into consideration and search for the data gathered from various databases was carried out by using a set of keywords including *B. pacumbis*; phytochemistry; kidney stone healer; anti-influenza; and antioxidant properties. In this paper, the literature has only been taken from the published work though some additional data may be taken from unpublished work, Ph.D. theses, etc. Additionally, the reported phytochemicals in this species were verified using the IUPAC name from PubChem; structural and chemical formulae were drawn and confirmed from Chem Draw software in ACS format and Pubchem, respectively. Furthermore, for reference, we used the software Mendeley.

### 2.2. Botanical Description

The botanical classification and Indian vernacular names of *B. pacumbis* are given in Table 1 [3,19,21,40].

### 2.3. Morphology

**Figure 1:**
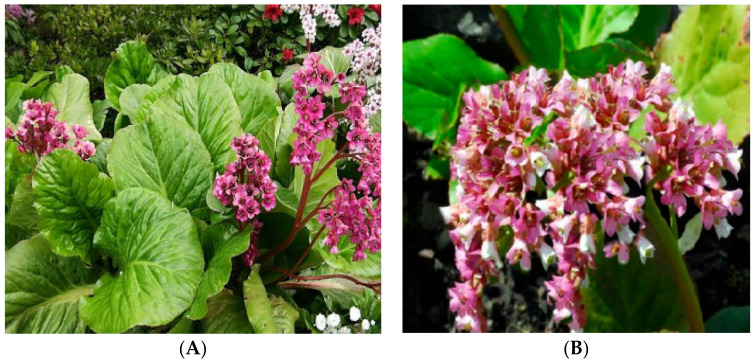
*Bergenia pacumbis* (Buch.-Ham. ex D.Don) C.Y.Wu & J.T.Pan (**A**) Whole Plant (**B**) Flower [3,13].

*Bergenia pacumbis* (Figure 1) is a rhizomatic perennial herb bearing leathery, rubbery, and fleshy leaves growing up to 30–35 cm tall, having a rhizomatous rootstock with intermittent auxiliary buds and a short stem [3,21,22,41]. The plant is very hardy and can survive throughout wintertime by turning red. It is an evergreen plant and has blooming flowers, which are bisexual with around 4–10 cm long cymose panicles in the period between April to June with characteristic white, pink, and purple. It has a surprising survival technique in which the rhizome arises out from the crevices of stones or rocks and hangs in the air on the slopes and can capture moisture from the air. Its leaves are glabrous, long, sparsely hairy in margins, widely obviate or elliptic, and finely or shallowly sinuate-dentate. The fruit is contained around a capsule-like structure containing numerous minute grey seeds in each capsule [3,22,40,41,42].

### 2.4. Distribution

**Figure 2:**
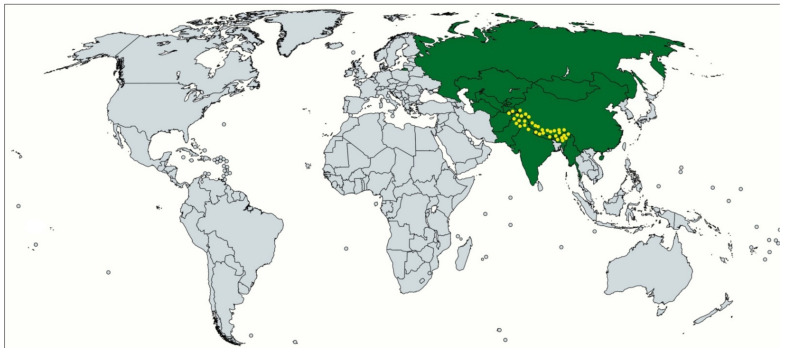
Geographical distribution of the genus *Bergenia* (in green) and *Bergenia pacumbis* (Buch.-Ham. ex D.Don) C.Y.Wu & J.T.Pan (with yellow dotes, mainly the Himalayan region) [3,13,42].

In India, the plant is found mainly in the northeast temperate Himalayas particularly in Jammu & Kashmir, Uttarakhand, Himachal Pradesh, and northeastern hilly states between the altitudes of 1200–3000 m, in rocky slopes of stone crevices present in cold or glacial mountains (Figure 2). It is also reported at higher altitudes in the Himalayan area of adjoining countries of Indian such as Afghanistan, Pakistan, Nepal, China, and Tibet [3,21,40,42].

### 2.5. Agro-Techniques

#### 2.5.1. Cultivation and Soil Conditions

*Bergenia pacumbis* grows well in temperate and humid climatic environments, where the temperature usually remains less than 20 °C. The plant grows healthy in sandy soil, which is somewhat acidic, has higher porosity, and is richer in humic organic matters. Owing to its hardy nature, this species can also grow well in medium loamy to clay soils that is augmented with manure. It bears light shade and raises well beneath open sunny environments. Nevertheless, vegetative growth is better in the shade, which takes about one month to develop to supply planting material for raising cultivation. So, the *B. pacumbis* is not suitable for sunny areas but grows well in shady and moist areas [43].

#### 2.5.2. Propagation by Rhizome

For faster regeneration of the species in the field during late summer or the onset of monsoons, the plant can be raised directly by planting 7.5–12.5 cm long rhizome segments with 2–3 nodes as propagation material. Raising crops by the aforementioned technique can lessen the crop cycle by one year in comparison to traditional method of propagation (by seeds). However, numerous rhizome segments have been required for planting to have faster regeneration. For this purpose, smaller rhizome segments (2 cm thick) can be imbedded in the soil at a spacing of 10 × 10 cm. The growing rate of the species is slow and it takes around 18 months to raise the species in the nursery for field imbedding [43,44].

#### 2.5.3. Propagation by Seeds

The second method is using seeds that are tiny in size and show poor viability and propagation potential. The seeds are bedded at 4 °C for 15 days to enhance germination. The storing of seeds leads to a drop in viability of the seeds. Greenhouse conditions are needed for better results [43,44]. The seeds take around 60–90 days to germinate. After germination, the seedlings are ready to plant in fresh nursery beds once it reaches the two-three leaved stage. The seedlings are planted at a spacing of 10 × 10 cm. It takes a whole season to grow large enough for imbedding in the field [43,44].

## 3. Traditional Uses

*Bergenia pacumbis* is used to treat various ailments by the Indian people, referenced in ancient medicinal books of India. The literature revealed that the plant has been used to treat urinary diseases and many other ailments since ancient times (Table 2).

From the prehistoric time, *B. pacumbis* water-extract has been usually used to cure kidney-stone and urogenital disorders. In Nepal, the paste prepared from *B. pacumbis* rhizome is consumed as a remedy to treat various diseases including ulcers, diarrhoea, colds, dysuria, coughs, pulmonary infusions, spleen enlargements, and fevers. The rhizomes along with molasses are also consumed to remove intestinal worms. In India, the dried roots of the plant are used to treat wounds, boils, burns, and ophthalmia [3,21,45,46,47]. *Bergenia pacumbis* possesses a vast range of disease curing properties but its full potential is still to be discovered. Literature has covered some traditional uses of this plant in its native habitats, and these are shown in Table 3. An extensive survey of the literature indicates that the plant is used not only to treat urinary diseases but also to treat many other ailments since ancient times and these are presented in Table 4. Though there are many traditional uses of *B. pacumbis* from the ancient days, researchers have currently manufactured some modern medicines using organic solvent extracts of various parts of this plant, which are described in Table 5.

## 4. Phytochemical Composition

**Figure 3:**
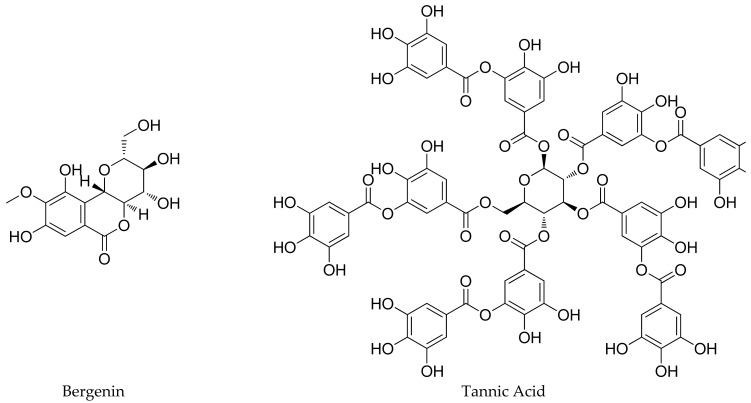

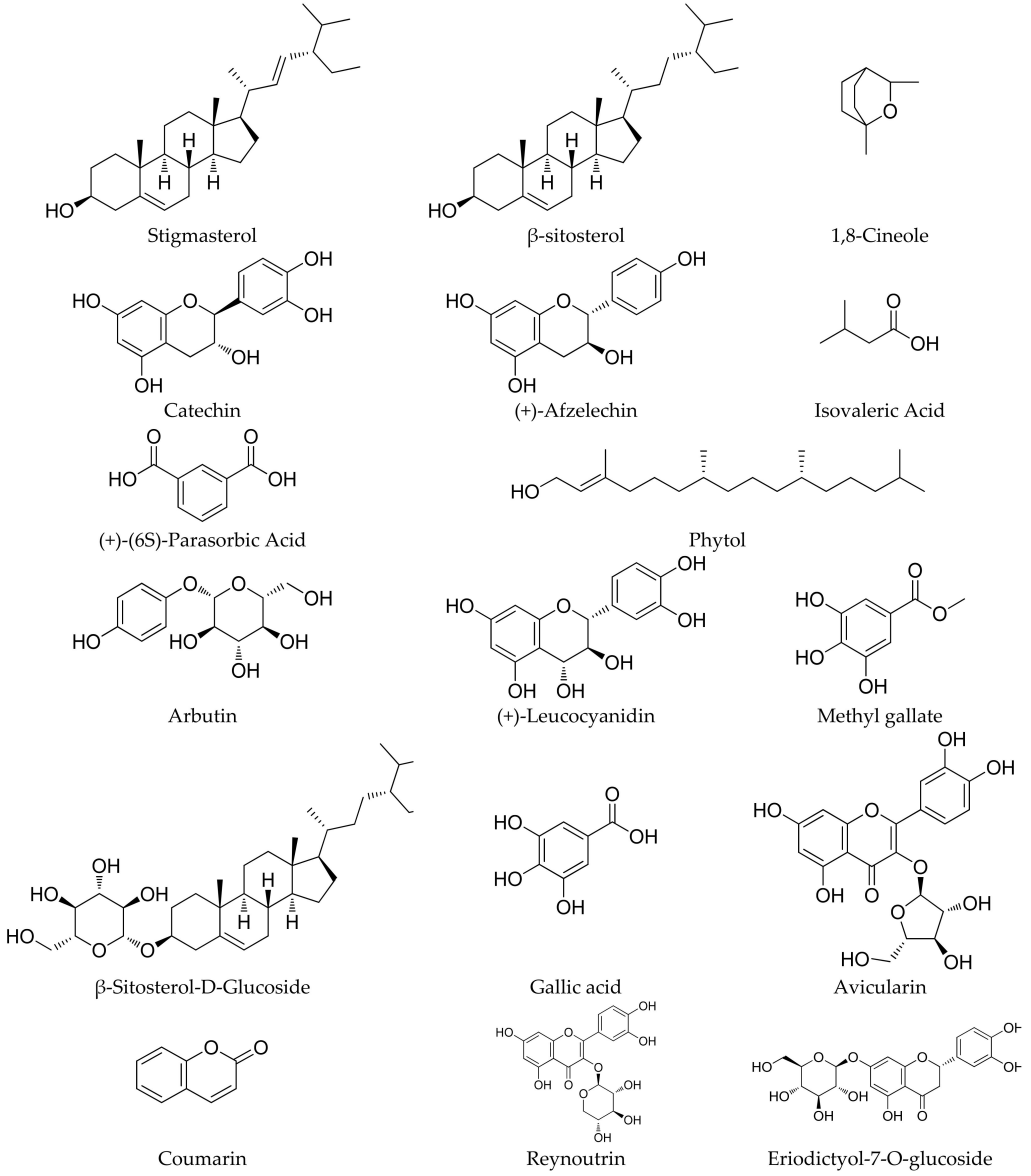

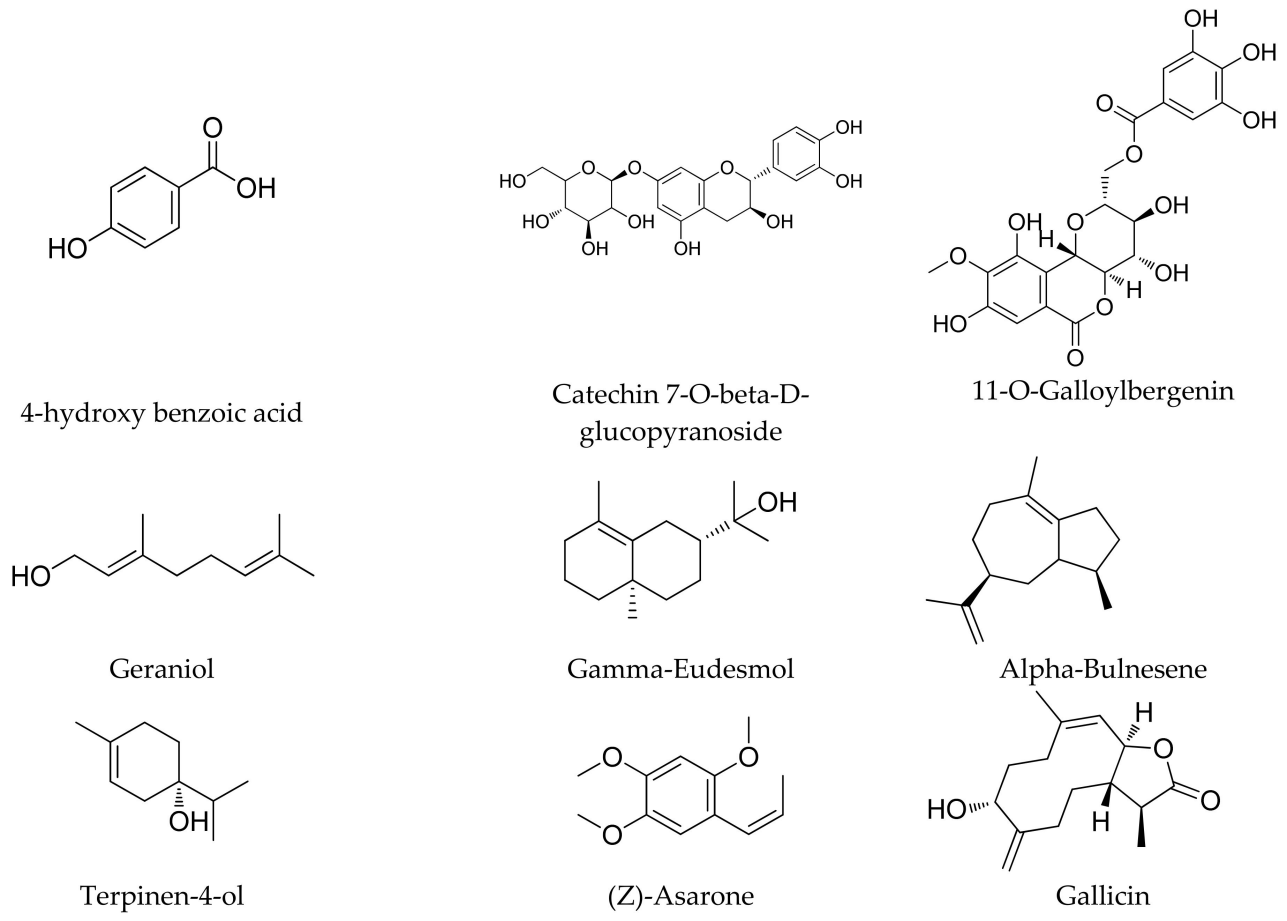
Phytochemicals present in *Bergenia pacumbis* (Buch.-Ham. ex D.Don) C.Y.Wu & J.T.Pan.

The presence of a diverse range of phytochemicals (Figure 3) imparts significant medicinal importance to *B. pacumbis*. Different phytochemicals are distributed all over the plant, as discussed in the following sub-sections. The qualitative analysis of various extracts obtained from different parts of *B. pacumbis* revealed the presence of tannins, flavonoids, quinines, phenols, carbohydrates, glycosides, cardio glycosides steroids, proteins, and saponins [3,21,42,59].

### 4.1. Phytochemicals Present in the Roots

The roots of *B. pacumbis* are rich in alkaloids, steroids, terpenoids, glycosides, and saponins. β-Sitosterol, stigmasterol, tannic acid, and gallic acid have been evaluated both quantitatively and qualitatively from roots using column and thin layer chromatography [21,60]. Roots were also rich source of various volatile bioactive constituents such as Terpinen-4-ol, (Z)-asarone, 1,8-cineole, isovaleric acid, and (+)-(6*S*)-parasorbic acid (47.45%) and were the major volatile phytoconstituents present in the essential oil [20,21].

### 4.2. Phytochemicals Obtained from the Rhizome

The rhizome contains many phytochemicals such as paashaanolactone, arbutin, (+)-afzelechin, bergenin, catechin, leucocyanidin, methyl gallate, ß-sitosterol-D-glucoside, glucose, avicularin, eriodictyol-7-ß–D-glucopyranoside, reynoutrin, 11-O-galloyl bergenin, 11-O-brotocatechuoyl bergenin, 4–0-galloyl bergenin, 6-O-p-hydroxy-benzoyl arbutin, 6-oprotocatechuoyl arbutin, 4-hydroxy benzoic acid, idehcxan-5-olide, catechin-7-O-ß–D-glucopyranoside, gallic acid, and oxalic acid [3,21,61]. Apart from these, rhizomes are also rich source of minerals, vitamins, albumin, glucose, mucilage, and starch [3,21,33].

### 4.3. Phytochemicals Isolated from Seeds

Seeds mainly contain coumarin, gallic acid, tannic acid, wax, and minerals [21,61].

### 4.4. Quantitative Analysis Phytoconstituents Present in B. ligulata

The result of quantitative analysis of *B. pacumbis* revealed the presence of total phenol, flavonoid, and tannin in amounts of 139.8 ± 9.06 mg of gallic acid equivalent/g, 77 + 6.40 mg of quercetin equivalent/g and 70.4 + 6.40 mg of tannic acid equivalent/g of plant extract at 500 μg/mL concentration [59].

Dhalwal and coworkers carried out the quantitative analysis of gallic acid, (+)-catechin, and bergenin from *B. pacumbis* by a HPTLC method. All the results were represented as % *w*/*w*. The result revealed that the bergenin (rhizomes—0.791 ± 0.014, petiole—0.090 ± 0.022, and leaf—0.115 ± 0.010) had the highest concentration followed by (+)-catechin (rhizomes—0.070 ± 0.017, petiole—0.021 ± 0.012, and leaf—0.009 ± 0.014), and gallic acid (rhizomes—0.030 ± 0.017, petiole—0.007 ± 0.032, and leaf—0.010 ± 0.012) [62].

Dharmender and coworkers carried out the quantitative analysis of β-sitosterol, gallic acid, gallicin, (+)-catechin, and bergenin from *Bergenia ciliata* (Haw.) Sternb. forma *ligulata* Yeo. All the results were represented as % *w*/*w*. The result revealed that the bergenin (0.11 ± 0.0026) had the highest concentration followed by gallicin (0.048 ± 0.007), β-sitosterol (0.044 ± 0.031), (+)-catechin (0.036 ± 0.003), and gallic acid (0.022 ± 0.002) [63].

### 4.5. Essential Oils

The essential oil obtained from the roots of *B. pacumbis* contained a large number of bioactive volatile phytoconstituents. Terpinen-4-ol (2.96%), (Z)-asarone (3.50%), 1,8-cineole (4.24%), isovaleric acid (6.25%), and (+)-(6*S*)-parasorbic acid (47.45%) were the major volatile phytoconstituents present in the essential oil. The other minute phytoconstituents identified in the essential oil were pentanol, perilla ketone, carvone-2-hexanal, *β*-pinene, limonene, santene, linalool, piperitone, ethyl acetate, carvacrol, geraniol, thymol, styrene, *p*-cymene, cadalene, myrtenol, menthol, elemol, heptanol, *R*-terpinene, benzaldehyde, furfural, 2,4,6-trimethylphenol, *R*-humulene, perilla aldehyde, geranyl acetone, hexanol, 4-vinylguaiacol, phenyl acetaldehyde, linalool oxide, hexanoic acid, seychellene, Γ-terpinene, (*E*)-anethole 9 *R*-pinene, nonanoic acid, 6-methyl-5-hepten-2-one, sabinene, (2*E*,4*Z*)-decadienal, guaiacol, *p*-mentha-2,4(8)-diene, 2-pentylfuran, R-calacorene, benzyl alcohol, δ-hexalactone, *γ*-nonalactone, dihydroaromadendrane, isoledene, *β*-patchoulene, camphor, *β*-eudesmol, humulene epoxide, (*Z*)-methylisoeugenol, neryl propionate, hrmsoia lactone, *R*-bulnesene, Ar-curcumene, acetophenone, camphenone, muurolene, phenylethanol, *β*-ionone, kessane, dihydroturmerone, pinocarvone, γ-eudesmol, (*Z*)-asarone,cis-pmenth-2-en-1-ol, isophorone, 1,3,5-trimethylnaphthalene, nonanal, 9-methyl-9H-fluorene, octanol acetate, dodecanai, *R*-terpineol, veratrole, patchouli alcohol, 2-(methylthio)ethanol, (2*E*)-nonen-1-al, (3*Z*)-butylidene phthalidey, borneol, *p*-cymen-8-ol,phenanthrene, trans-calamenene, (*E*)-asarone, palmitic acid, methyl salicylate, *R*-muurolol, and cumin aldehyde [20,21].

## 5. Pharmacology

**Figure 4:**
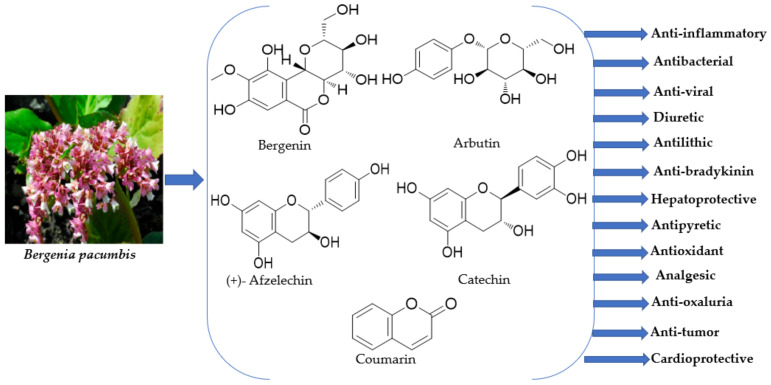
Critical pharmacological applications of *Bergenia pacumbis* (Buch.-Ham. ex D.Don) C.Y.Wu & J.T.Pan.

Owing to the occurrence of a diverse range of phytochemicals, *B. pacumbis* shows numerous pharmacological activities. Till date, a diverse array of pharmacological activities such as anti-inflammatory, antibacterial, anti-viral, diuretic, antilithic, anti-bradykinin, hepatoprotective, antipyretic, α-glucosidase activity, free radical scavenging, analgesic, anti-oxaluria, anti-tumour, and cardioprotective activities have been reported from various parts of *B. pacumbis* [3,18,19,21,22,42] (Figure 4).

### 5.1. Anti-Inflammatory

Studies have exposed that aqueous and alcoholic extracts of fresh rhizomes of *B. pacumbis* show anti-inflammatory activity in biological systems at a dose level of 1 gm/kg. [64]. An amount of 0.1 mL of 1% carrageenan solution was injected into the left-hand paw of the rat and caused an increase in the volume of the rat’s paw. This volume increment is measured every hour, and then the inhibition percentile is calculated. Results show that *B. pacumbis* has an excellent potential for anti-inflammatory activity [64,65]. Research has also revealed that *B. pacumbis* possesses some radical scavenging activity [66].

### 5.2. Antibacterial

The literature revealed that *B. pacumbis* extracts also possess antibacterial activity [3,21,64,67]. The plant extract was used in three concentrations (10, 25, 50 mg/mL), and the antibacterial activity was measured via the disc diffusion method. The various extracts of the plant (methanolic, ethanolic, and aqueous) was tested in a culture plate containing *Escherichia coli, Bacillus subtilis,* and *Staphylococcus aureus* at the dosages mentioned above and the extracts contain significant antibacterial activities. Reports show that at a concentration of 50 mg/mL, the antibacterial activity reached maximum levels, which were found to be equal to the antibacterial activity of ciprofloxacin (25 mg/mL) [64].

### 5.3. Anti-Viral

Anti-viral activities of *B. pacumbis* have been reported in a study on Nepalese medicinal plants [35,36]. The extracts (methanolic and hydromethanolic) were analyzed for influenza and herpes viruses, and the highest anti-influenza viral activity was observed at the dosage of 10 μg/mL [68]. The rhizome of *B. pacumbis* was used to prepare an extract containing methanol and water as a solvent, and this extract had good viral inhibitory properties against the influenza virus [21,68]. The extract inhibits the viral RNA synthesis, and the study shows that the peptide synthesis rate was decreased strongly at the concentration of 10 μg/mL. The study revealed that tannin is the main component present in the plant rhizome extract, increasing protein availability and acting as an antioxidant and as a metal ion chelator in the chosen biological systems [22].

### 5.4. Diuretic Activity

The ethanolic extracts of *B. pacumbis* roots were tested on albino rats to study their diuretic activity using the Lipschit method [13,69]. Diuretic activity was suspected by measuring the volume of urine collected at an interval of 5 h and also by measuring the Na^+^, K^+^, and Cl^−^ ion concentrations in urine collected from the rats [70]. The ethanol extracts possess the highest diuretic activity. The same group of researchers also studied the effects of an ethanolic extract of *B. pacumbis* roots on artificial urine and human urine where CaC_2_O_4_ crystals were introduced in the first one. In the case of human urine, the crystals were already present. On adding extract prepared from the roots of *B. pacumbis*, artificial urine showed the reduction of the crystal ring size, which confirms that the extract may be active in-vitro. Nevertheless, when the extract was applied to the human urine, it showed remarkably other characteristics than CaC_2_O_4_ crystal inhibition such as antioxidant effects and hypermagneseuric effects. From these results, it was concluded that *B. pacumbis* possesses diuretic activity [64,69,70].

Further studies revealed that methanolic extracts of *B. pacumbis* and bergenin showed a noticeable dissolution of urinary calculi in the kidney. In-vitro antilithiatic/anti calcification potential of different extracts obtained from *B. pacumbis* and *Dolichos biflorus* L. were tested independently and in combination by the homogeneous precipitation method [33]. The results of tested extracts were compared against ‘Cystone’ (a Himalaya company formulation sold in India) aqueous extract. *Bergenia pacumbis* extract showed lesser activity while *D. biflorus* extract displayed almost equivalent activity as compared to ‘Cystone’. Although, the combination of two extracts is less active in comparison to the individual extracts. The author concluded that active constituent/s may act by inhibiting calcium and phosphate accumulation and are non-protein and non-tannin in nature. Another study on rats revealed that, the low doses of *B. pacumbis* alcoholic extracts (0.5 mg/kg) promote diuresis, but higher doses (100 mg/kg) retard the diuresis produced by urea and urine output. It is also reported that, the aqueous extracts of *B. pacumbis* have better potential as compared to the aqueous extract of *Tribulus terrestris* L. for inhibiting the growth of calcium oxalate monohydrate crystals [32]. This study showed that there are secondary metabolites present in *B. pacumbis* known to play a significant role in the dissolution of calcium oxalate monohydrate crystals [33].

### 5.5. Antilithic

The antilithic property of an alcoholic extract of *B. pacumbis* showed no effect in rats in the inhibition of stone formation, but the low dosage of crude alcoholic extract (0.5 mg/kg of extract) endorses the diuresis, and higher dosage (100 mg/kg of extract) reduced the diuresis produced by urea [71,72]. The study also revealed that applying 0.75% ethylene glycol in water (5–10 mg/kg extract) of *B. pacumbis* rhizome in rats prevents the deposition of the crystal in the renal tubules of a rat. The application of *B. pacumbis* rhizome extract prevented the side effects after lithogenic treatment such as polyuria, decreased antioxidant, weight loss, renal dysfunction, etc. The study also showed that after extract application, there was a slight increase in the Mg^2+^ ions in the urine, indicating the anti-urolithic activity of *B. pacumbis* [28,29,34,54,71].

### 5.6. Anti-Bradykinin Activity

Though the rhizome extract of *B. pacumbis* shows anti-bradykinin potential, it does not affect the activity of acetylcholine and 5-hydroxytryptamine (5-HT) on guinea-pig ileum. The rhizome extract increases the adrenaline level on the guinea pig trachea, and in addition, the smooth ileum muscle shows cardiotoxicity and central nervous system depressant activity. In rats, the lethal dosage of the aqueous extract of *B. pacumbis* rhizome was 650 mg/kg (i.p.). It is widely used to treat painful or difficult urination, renal failure, infection, or inflammation of the urinary bladder and crystalluria, which is caused due to the side effects of sulfonamides and penicillin, abscesses, cutaneous infection, dysentery, and diarrhoea [3,17,61].

### 5.7. Hepatoprotective

The hepatoprotective activity was investigated in albino rats (weight 25–35 gm) by using the ethanolic extract of *B. pacumbis* roots and compared with the standard drug “Liv-52” (manufactured by Himalaya Drug Company, Bangalore), by inducing hepatotoxicity using carbon tetrachloride (CCl_4_). The investigation was performed using the Up and Down or Staircase method [70]. The ethanolic extract of *B. pacumbis* restored the integrity of hepatocytes indicated by improvement in physiological parameters, which was confirmed by measuring the levels of transaminase, serum alkaline phosphatase, oxaloacetate, serum glutamate, pyruvate transaminase, serum glutamate, and bilirubin levels and known to have a significant hepatoprotective potential [3,13].

### 5.8. Antipyretic

**Figure 5:**
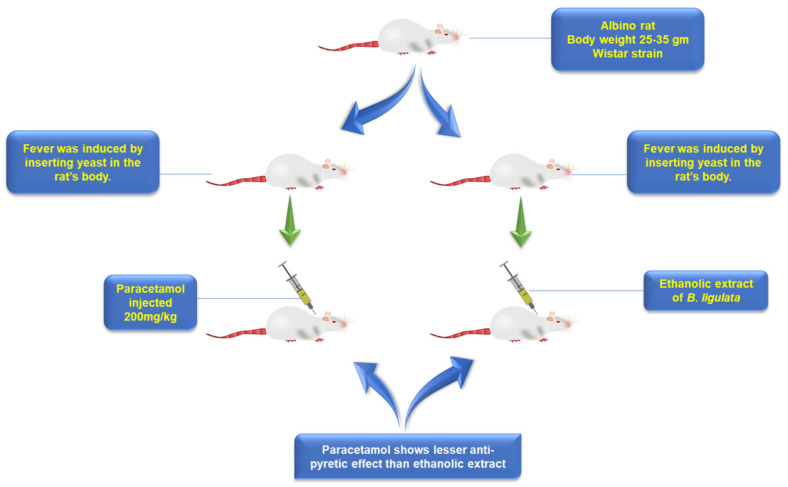
Antipyretic activity of *Bergenia pacumbis* (Buch.-Ham. ex D.Don) C.Y.Wu & J.T.Pan.

The literature revealed that *B. pacumbis* possess a substantial antipyretic potential. Singh and coworker examined the ethanol (95%) and aqueous extract obtained from the roots of *B. pacumbis* for their antipyretic potential. The extracts were mixed with 2% gum acacia and injected to Wistar rats (500 and 300 mg/kg body weight) infected with pyrexia [70]. Paracetamol (200 mg/kg, standard antipyretic) was used as a positive control. The rectal temperature of the infected rats was noted after an interval of 1 h. A noteworthy lowering in the rat’s body temperature was noted with ethanol extract (500 mg/kg) (Figure 5). The present study along with others reports validated that *B. pacumbis* owns substantial antipyretic activity [13,70,73].

### 5.9. α-Glucosidase Inhibition Activity

The ethanolic extract (80%) of *B. pacumbis* rhizome led to the investigation of α-glucosidase activity at dose levels of 5.0, 0.5, and 0.05 mg/mL and the ethyl acetate extract was used to inhibit the effect of α-glucosidase activity. The trigger compound was identified as (+)-afzelechin (2 g), which was confirmed by EI-MS, IR, proton NMR, and ^13^C NMR spectral analysis [29]. Further, the inhibitory activity of the compound at a concentration of 0.25 mM was studied at a 50% inhibition dose, i.e., 0.13 mm. ID_50_ values of (+)-catechin and (-)-epicatechin were 12.8 mM and 0.18 mM, respectively. From these data, the α-glucosidase inhibitor in *B. pacumbis* is primarily due to the presence of (+)-afzelechin [20,29,37].

### 5.10. Antioxidant Activity

*Bergenia pacumbis* methanolic extract efficiently scavenge 1, 1-diphenyl-2-picrylhydrazyl (DPPH) free radicals and exhibit good free radical scavenging potential with an IC_50_ value of 50 µg/mL. The water and n-butanol fractions obtained from methanol extract were screened for their free radical scavenging potential (in-vitro) by DPPH and the nitric oxide assay. The n-butanol and water fractions showed the IC_50_ value of 4.5 µg/mL and 30 µg/mL, respectively [48,74]. Bergenin isolated from *B. pacumbis* also showed significant antioxidant potential [59,75].

### 5.11. Analgesic

The analgesic potential of *B. pacumbis* rhizomes was assessed by employing hot plate and tail clip methods using hydroalcoholic extract (250 mg/kg), which was administered intra-gastrically in the mouse. However, the extract exhibited much less analgesic potential during the study [3,13,22].

### 5.12. Anti-Oxaluria

Anti-oxaluria activity on Indian adults was studied where tablets were prepared with *Didymocarpus pedicellatus* R.Br., *B. pacumbis, Rubia cordifolia* L., *Cyperus scariosus* R.Br.*, Achyranthes aspera* L., *Veronica cinerea* Boiss. & Balansa, Hajrul yahood bhasma, and Shilajeet purified in the ratio 65:49:16:16:16:16:16:13 (in mg) and investigated on 32 healthy volunteers and 48 people suffering from stones. All patients were given two tablets (3 times/day) and advised to avoid oxalate-rich foods in their diet, and the treatment lasted for 8 weeks. A steady decrease in oxalate elimination was noted in persons infected with kidney stones, but the level of oxalate elimination was not as low as observed in usual adults. This study revealed that the present formulation might deliver a capable drug that regulate the activity of oxaluria [3,76].

### 5.13. Anti-Tumor

*Bergenia pacumbis* hydroalcoholic extract was injected intraperitoneally in rats to evaluate its anti-tumour potential. The extract exhibited activity against SARCOMA WM1256 IM cell culture at the dose of 20 mcg/mL, which pointed out that *B. pacumbis* hydroalcoholic extract exhibited cytotoxic activity [3].

### 5.14. Cardioprotective

The hypotensive activity of *B. pacumbis* hydroalcoholic extract was carried out in different animal models. A positive hypotensive activity was noted in dogs when injected with 50 mg/kg dose (i.v.) [3]. Further, the *B. pacumbis* extract also showed positive inotropic and chronotropic effects on a frog’s heart [17]. While in the case of continuous rabbit’s heart perfusions, the extracts exhibited adverse chronotropic and inotropic effects with a decrease in coronary flow. The alcoholic extract *B. pacumbis* elicited marked anti-bradykinin activity (in-vitro and in-vivo) but was unable to modify the response of acetylcholine and 5-HT on guinea-pig isolated ileum [3,77].

### 5.15. Insecticidal Activity

Kashima and coworkers evaluated the insecticidal potential of essential oil and parasorbic acid obtained from roots of *B. pacumbis* against adults of *Drosophila melanogaster*. The results revealed that both the essential oil and parasorbic acid were active against the insect, but parasorbic acid had more insecticidal potential as compared to the essential oil [20].

## 6. Patents

Mitra has filed a patent on a skincare product maintaining the skin around the eyes, prepared from the extracts of *B. pacumbis*, *Emblica officinalis* Gaertn., *Cipadessa baccifera* (Roxb. ex Roth) Miq., and cosmeceutically acceptable constituents [3,67]. Lee and Martin patented a skincare product to maintain skin and tanning [67,78]. Agarwal and Kumar also patented a better and improved process for isolating the main component, bergenin, from *Bergenia* species [3,79].

## 7. Safety and Toxicity Profile

*Bergenia pacumbis* is primarily used as one of the critical ingredients of various preparations for the treatment of kidney diseases, e.g., Nephrolex and Cystone (Himalaya Herbal Healthcare, Bangalore). The acetone extract obtained from *B. pacumbis* rhizomes is cardiotoxic in nature at higher dosages. It is also reported to have a depressant or sedative effect on the CNS (central nervous system) [3]. The CNS depressant, antidiuretic and cardiotoxic effect of *B. pacumbis* on experimental models have been noted only at higher dosages. The LD_50_ of the aqueous extract in rats has been found to be 650 mg/kg when injected intraperitoneally [3,77].

**Dosage:** For decoction: 20–30 gm rhizomes; Powered rhizomes: 1–3 gm twice a day [3,77,80].

## 8. Prospects and Medicinal Opportunities

*Bergenia pacumbis* is one of the essential folk medicinal herbs found in the Himalayan region. It is primarily used for coughs, to stop bleeding, and to increase immunity [3,21]. Among the numerous phytochemicals present in this plant, the polyphenols are mainly of interest, showing a wide range of medicinal properties.

Edible and Cosmetic: *Bergenia pacumbis* possesses many minerals and amino acids, which show antibiosis and dieresis properties and may be used as a disinfectant of urine. This study was performed both in-vivo and in-vitro on male Wistar rats to understand better the medicinal use of the various extracts of *B. pacumbis* [34].

For cosmetic aspects, the presence of arbutin in the plant can make a skin brightening agent because it causes inhibition of tyrosinase on the skin. Also, the presence of so many phenolic compounds implies a potential way to synthesize metallic nanoparticles, as literature has revealed that nanoparticle synthesis is possible from the extracts of other species of the *Bergenia* genus [81].

Gaurav and Gaurav raised various authentication, overexploitation, and standardization as drug pharmacology in their review article on *B. pacumbis* published in 2014 [3]. The present article addressed all these issues and will be very helpful for proper authentication, identification, cultivation, conservation, and utilization of *B. pacumbis*.

## 9. Conclusions

Much research has been performed on the *B. pacumbis* plant during the last few decades to investigate its phytochemical composition, biological potential, and traditional and modern uses. The present review reports recent information about medicinal uses, phytochemicals, biological investigations, and patents of *B. pacumbis*. *Bergenia pacumbis* is a highly valued medicinal plant and has been traditionally used among the various communities across India, Pakistan, Nepal, and Tibet, mainly in the Himalayan region to treat urinary disorders, respiratory problems, influenza, asthma, and inflammatory and infectious diseases. Further, rhizomes and leaves of the species are also used to treat kidney stones and various other disorders associated with the kidney. Almost all parts of *B. pacumbis* are used to treat various ailments, but the most used part is the rhizome. The preferred mode of utilization is powdered drug and aqueous extract of rhizome and leaves. Pharmacological investigations showed that it has potent anti-urolithic, antioxidant, anti-viral, free radical scavenging, antidiabetic, hepatoprotective, diuretic, antipyretic, anti-oxaluria, anti-tumour, antibacterial, anti-fungal, anti-inflammatory, antimicrobial, and cardioprotective potential.

It is quite evident from present review that the *B. pacumbis* is known to have a wide range of bioactive phytochemicals that possesses tremendous therapeutic values. The efficacy and safety of different parts of *B. pacumbis* have been well recognized and time-tested during the prolonged historical traditional uses. However, still there are many pharmacological activities that are yet to be explored. In addition, there is a tremendous scope of research related to the investigations about the mechanisms of action of the various aforementioned pharmacological activities. Further, the toxicity and safety of *B. pacumbis* have not been explored much, so further work is also required in this particular domain.

The current review suggests a varied opportunity for several benefits of *B. pacumbis* in the field of pharmaceuticals, health foods, cosmetics, floriculture, and many other economic and industrial endeavors. The present study can be beneficial in proper authentication, identification, cultivation, conservation, and utilization of *B. pacumbis* and may contribute to the direction of further scientific research to explore more about its pharmacology, toxicology, cultivation techniques, conservation, and bioactive formulations. To conclude, *B. pacumbis* has a vast potential to act as a remedy to various health-related illnesses, and thus its conservation is requisite.

## Figures and Tables

**Table 1:** Botanical and vernacular names of *Bergenia pacumbis* (Buch.-Ham. ex D.Don) C.Y.Wu & J.T.Pan.

Botanical Classification [3,19,21,22,40]	Vernacular Names [3,21,22]
**Kingdom:** Plantae **Sub Kingdom:** Tracheobionta/vascular plant **Super Division:** Spermatophyta/seed plants **Division:** Magnoliphyta/Flowering plants **Class:** Magnoliopsida/Dicotyledons **Sub Class:** Rosidae **Order:** Rosales **Family:** Saxifragaceae **Genus:** *Bergenia* Moench-elephant ear **Species:** *Bergenia pacumbis* (Buch.-Ham. ex D.Don) C.Y.Wu & J.T.Pan	**Sanskrit:** Pashaanbheda, Silabheda, Nagbhita, Ashmabheda **Hindi:** Dakachru, Pakhanabhede, Pakhanabheda **Assamese:** Patharkuchi **Mizuram:** Khamdamdawi, Pandamdawi **Punjab:** Dharposh, Batpia, Pashanbhed, Kachalu **Bengali:** Himasagara, Patharchuri **Tamil:** Sirupilai **Telugu:** Condapindi, Telanurupindi **Urdu:** Pakhanabheda, Kachalu

**Table 2:** Traditional uses of *Bergenia pacumbis* (Buch.-Ham. ex D.Don) C.Y.Wu & J.T.Pan highlighted in different books related to Indian traditional medicinal systems [3,21,22,45,46,47].

Indian Traditional Medicinal Systems and Related Books	Traditional Uses
Ayurveda	Ayureveda documented the use of leaf extract of *B. pacumbis* to treat various urinary disorders such as stone formation. It is also used to treat hemorrhagic disease, stomach related pain, and neurological disorders that cause seizures or unusual sensations and behaviour.
Sushruta Samhita	Sushruta Samhita highlighted the use of plant extract to dissolve kidney stones, inhibit stone formation, also help to treat sugar-related problems.
Charak Samhita and Chakradatta	Charak Samhita and Chakradatta revealed the use of *B. pacumbis* mainly for treatment of urinary diseases.
Unani	Unani system of medicine documented the potential of *B. pacumbis* in stone dissolution.
Rajnighantu	Acoording to Rajnighantu *B. pacumbis* is mainly used for the treatment of various ailments associated with urinary bladder.
Bhavaprakash	Prevention of causing the contraction of skin cells and other body tissues, also helps to treat urinary related problems.

**Table 3:** Traditional uses of *Bergenia pacumbis* (Buch.-Ham. ex D.Don) C.Y.Wu & J.T.Pan in different region [21].

Location	Usable Parts	Traditional Use
Uttar Pradesh	Root	Boils, cuts, wounds, and ophthalmia, kidney stones, urinary complaints
Johari (Iqbal Tehsil of Lahore, Punjab, Pakistan)	Root	In asthma, urinary troubles [48]
Kumaoni	Rhizome	In fever and thirst
Monpa (Arunachal Pradesh)	Leaf	In boils, cuts, wounds [49]
Naga	Root	In liver complaints and TB [50]
	Leaf	Boils, cuts, and wounds
Central Himalaya Region	Plant	In dizziness, headache, vertigo
	Leaf	For dissolving kidney stones [51]

**Table 4:** Method of uses of *Bergenia pacumbis* (Buch.-Ham. ex D.Don) C.Y.Wu & J.T.Pan by various tribes and local people [21].

Types of Diseases	Method of Use
Dissolution of kidney and gall bladder stone	Rhizome extract is dried and then swallowed [52]
Wound healing	Powder of dried leaves and rhizomes are applied to heal old wounds
Cough and cold	Leaves and rhizome are boiled with water and swallowed [53]
Cuts and burns	Crushed rhizome is mixed with curd and applied gently on burns [27]
Dysentery and diarrhoea	Extract of rhizome is taken orally
Fever	Rhizome is dried and taken orally
Asthma	Rhizome juice is taken orally [54]
Gastro-intestinal problems	Fresh rhizome chewed
Eye ailments	Crushed fresh rhizome should sap on the eye
Chronic ulcer	Rhizome extract should be taken orally [55]
Inflammation, rheumatic, helmintic, piles, tonsils, aphrodisiac, colitis, cardiac problems, urinary diseases	Rhizome and leaves extract are dried well and should be taken orally [56]

**Table 5:** Modern uses of various extracts obtained from *Bergenia pacumbis* (Buch.-Ham. ex D.Don) C.Y.Wu & J.T.Pan.

Ailments	Solvent Extract
Antilithic activity	Alcoholic extract of *B. pacumbis* root [56]
Anti-inflammatory, Cardiotoxic, CNS depressor	Acetone extract of *B. pacumbis*
Anti-diuretic activity	High doses of acetone extract [57]
Spasmogenic activity, anti-protozoan, anti-cancer	Ethanolic extract of rhizome [58]
Anti-glucosidase, anti-pyretic, diuretic, Hepatoprotective, anti- cancer, anti-protozoan, cardiovascular, anti-scorbutic, anti-lithiatic, anti-viral.	Different parts of *B. pacumbis* [4]
